# Supplementary material for: High-pressure synthesis of ultraincompressible hard rhenium nitride pernitride Re2(N2)(N)2 stable at ambient conditions
Source: Nat Commun. 2019 Jul 5;10:2994. doi: 10.1038/s41467-019-10995-3 (PMC6611777; doi:10.1038/s41467-019-10995-3)
Supplement: Supplementary file 1 — Supplementary information [file 41467_2019_10995_MOESM1_ESM.pdf]

High-pressure synthesis of ultraincompressible hard rhenium nitride pernitride $Re_2(N_2)(N)_2$ stable at ambient conditions

Maxim Bykov et al.

Supplementary Note 1. Analysis of multigrain/multiphase datasets

If a chemical reaction occurs in a DAC during laser heating, it often leads to multiple domains of well-crystallized phase(s). Moreover, the diffraction spots originating from different grains not often overlap with each other and this allows to integrate the dataset separately for each grain. The same happened in a case of the Re – N_2 system. For the unit cell determination, we use the reciprocal space viewer (Ewald explorer) from *CrysAlis^{Pro}*. It allows to manually select the reciprocal lattice of the grain. Below we provide an example for the typical dataset of ReN_2. The Supplementary Figure 1 demonstrates the reciprocal space reconstruction with seven strongest lattices corresponding to the grains of ReN_2 in different orientations. The Supplementary Table 1 shows typical statistics for total/overlapped reflections in our datasets.

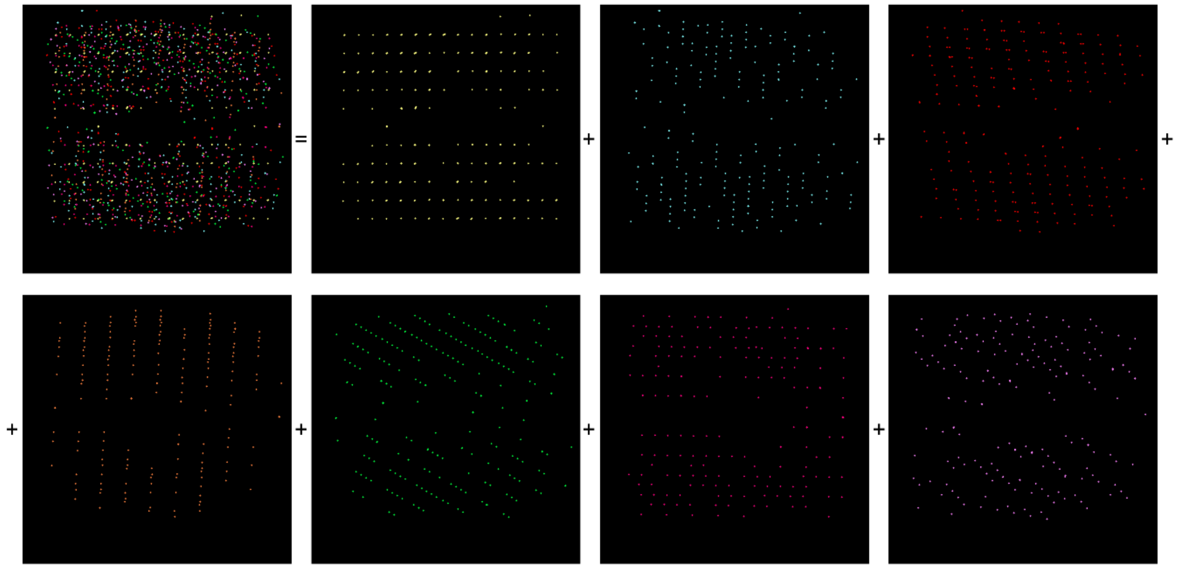

Supplementary Figure 1. Reciprocal space reconstruction with seven strongest lattices.

Supplementary Table 1. Typical reflection statistics for several ReN_2 grains, producing the strongest diffraction spots.

Grain	*a*, Å	*b*, Å	*c*, Å	*β*, °	*V*, Å^3	Reflections total / separate / overlapped
#1	3.5065	6.1482	4.6884	111.175	94.3	249/249/0
#2	3.5065	6.1544	4.6865	111.253	94.3	218/216/2
#3	3.5072	6.1467	4.6921	111.197	94.3	249/246/3
#4	3.5022	6.1615	4.6885	111.099	94.4	196/194/2
#5	3.5020	6.1620	4.6906	111.214	94.4	243/237/6
#6	3.5031	6.1528	4.6933	111.212	94.3	198/190/8
#7	3.4967	6.1539	4.6939	111.149	94.2	208/199/9

Supplementary Table 2. Details on the refinement of the crystal structure of ReN_2 at ambient conditions

**Crystal data**	
Chemical formula	ReN_2
M_r, g/mol	214.22
Crystal system, space group	Monoclinic, $P2_1/c$ (No. 14)
Temperature (K)	293
Pressure (GPa)	0.0001
a, b, c (Å)	3.6254(17), 6.407(7), 4.948(3)
β (°)	111.48(6)
V (Å^3)	106.96(15)
Z	4
Radiation type	Synchrotron, λ = 0.2903 Å
μ (mm^{-1})	10.76
Crystal size (mm^3)	0.03 × 0.02 × 0.01
**Data collection**	
Diffractometer	13IDD, APS, Chicago, USA
Absorption correction	Multi-scan
T_{min}, T_{max}	0.386, 1.000
No. of measured, independent and observed [$I > 2\sigma(I)$] reflections	290, 180, 175
R_{int}	0.011
$(\sin \theta/\lambda)_{max}$ (Å^{-1})	0.879
**Refinement**	
$R[F^2 > 2\sigma(F^2)]$, $wR(F^2)$, S	0.035, 0.088, 1.10
No. of reflections	180
No. of parameters	19
$\Delta\rho_{max}$, $\Delta\rho_{min}$ (e Å^{-3})	2.83, -2.67
**Refined crystal structure**	
Re (x, y, z)	0.35490(11), 0.34041(8), 0.19965(8)
N1 (x, y, z)	0.194(2), 0.038(2), 0.311(19)
N2 (x, y, z)	0.259(3), 0.6381(18), 0.024(2)
**Calculated crystal structure**	
Re (x, y, z)	(0.35397, 0.33961, 0.19931)
N1 (x, y, z)	(0.1889, 0.037, 0.30)
N2 (x, y, z)	(0.2540, 0.6397, 0.0164)

Supplementary Table 3. Selected geometric parameters of ReN_2 at ambient conditions (Å)

Re01—Re01^{i}	2.7318 (16)	Re01—N2^{vi}	2.095 (10)
Re01—Re01^{ii}	2.7318 (16)	N1—Re01^{iv}	2.112 (11)
Re01—N1	2.105 (12)	N1—Re01^{ii}	2.082 (9)
Re01—N1^{iii}	2.112 (11)	N1—N1^{vii}	1.412 (16)
Re01—N1^{i}	2.082 (9)	N2—Re01^{v}	2.082 (10)
Re01—N2	2.072 (11)	N2—Re01^{vi}	2.095 (9)
Re01—N2^{iv}	2.028 (11)	N2—Re01^{iii}	2.028 (11)
Re01—N2^{v}	2.082 (10)		

Symmetry code(s): (i) *x*, −*y*+1/2, *z*+1/2; (ii) *x*, −*y*+1/2, *z*−1/2; (iii) −*x*+1, *y*+1/2, −*z*+1/2; (iv) −*x*+1, *y*−1/2, −*z*+1/2; (v) −*x*+1, −*y*+1, −*z*; (vi) −*x*, −*y*+1, −*z*; (vii) −*x*, −*y*, −*z*.

Supplementary Note 2. Estimation of the nitrogen content in the WC-type ReN_x

The ambient-pressure unit cell parameters and the unit cell volume of the WC-type ReN_x phase, obtained in the experiments #1 and #7 do not agree with our and with previous theoretical calculations if x = 1 (Supplementary Table 4). Furthermore, the density of WC-ReN phase with ideal 1:1 stoichiometry from theoretical calculations is in a good agreement with the N-content – density trend of the Re-N system (Supplementary Figure 2a). However, the experimental density, calculated with the assumption of 1:1 composition, is clearly higher than expected (Supplementary Figure 2a). In order to estimate the nitrogen content, we have calculated the unit cell volumes of ReN_x with x varying from 0.58 to 1 (Supplementary Table 4, Supplementary Figure 2b). In the studied composition range, the dependence of the unit cell volume of ReN_x linearly depends on the nitrogen content. The result of the linear fit, presented in the Supplementary Figure 2b was used to estimate the composition of ReN_x obtained in the experiments #1 and #6.

We could not reliably detect $ReN_{0.6}$ phase in the experiment #2, which was used for the measurement of the equation of state. We would like to note here that the volume change of $ReN_{0.6}$ between 0 and 42 GPa from the available single-crystal data is only -9.75%, which makes it a very incompressible compound as well. Several theoretical works were devoted to the ReN compound and it was predicted that the most stable structure of ReN is NiAs like structure.[1,2] We believe that the role of vacancies in the stabilization of ReN_x ($x \leq 1$) phases must be taken into account in further theoretical calculations.

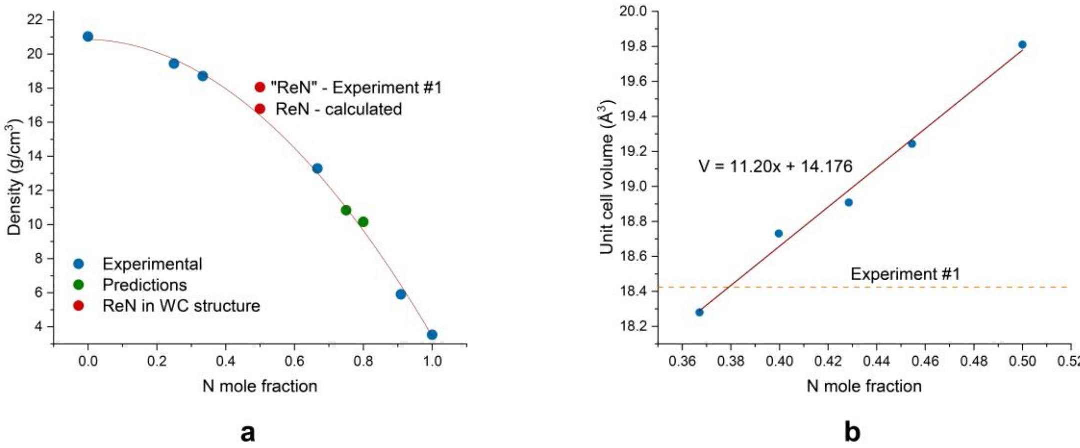

Supplementary Figure 2. (a) Density of Re-N compounds as a function of nitrogen content at ambient pressure. Blue points – experimental data on Re,[3] Re_3N,[4] Re_2N,[4] ReN_2 (this study), ReN_{10},[5] *cg*-N.[6] Green points – theoretically predicted compounds ReN_3 and ReN_4.[7] Red points – calculated and experimental density of ReN in WC structure type. (b) Unit cell volume of WC-type Re-N phase as a function of nitrogen concentration. Blue points – calculated volumes of $ReN_{0.58}$, $ReN_{0.666}$, $ReN_{0.75}$, $ReN_{5/6}$ and ReN. Red line – linear fit.

Supplementary Table 4. Calculated and experimental lattice parameters for WC-type structure of ReN_x at ambient pressure.

	V, Å^3	Method	Reference
**Experiments:**			
Experiment #1	18.424	SC XRD	This study
Experiment #6	18.19	SC XRD	This study
**Calculations:**			
ReN	19.46	LDA TB-LMTO	1
ReN	19.64	GGA-PBE	8
ReN	19.31	LDA, FP-LAPW	2
ReN	19.81	PBEsol	This study
$ReN_{5/6}$	19.24	PBEsol	This study
$ReN_{0.75}$	18.91	PBEsol	This study
$ReN_{0.666}$	18.73	PBEsol	This study
$ReN_{0.58}$	18.28	PBEsol	This study

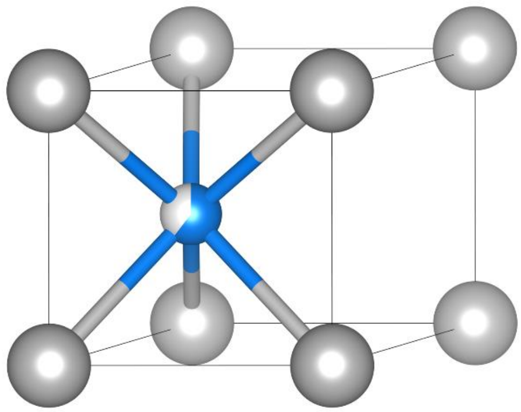

Supplementary Figure 3. The crystal structure of $ReN_{0.6}$ at ambient conditions. Re atoms – gray balls, N atoms – blue.

Supplementary Note 3. Representative powder diffraction patterns. Synthesis of ReN_2 *via* a reaction with nitrogen

Due to the small lattice parameters of defect WC-type ReN_x, we could not reliably detect it on some powder patterns, where it could be in a mixture with ReN_2 and Re_2N. Single-crystalline grains of ReN_x were found only in the experiments #1 and #6, but we cannot exclude that this phase may be present in other syntheses.

Some powder diffraction patterns contain very weak non-indexed peaks. These peaks may originate from the pressure-transmitting medium (N_2) or other minor phases. Le Bail fits were performed on the major phases, which have also been confirmed by the single-crystal experiments. Peak indexing of weak unknown phases based on the powder pattern in such a mixture would be unreliable.

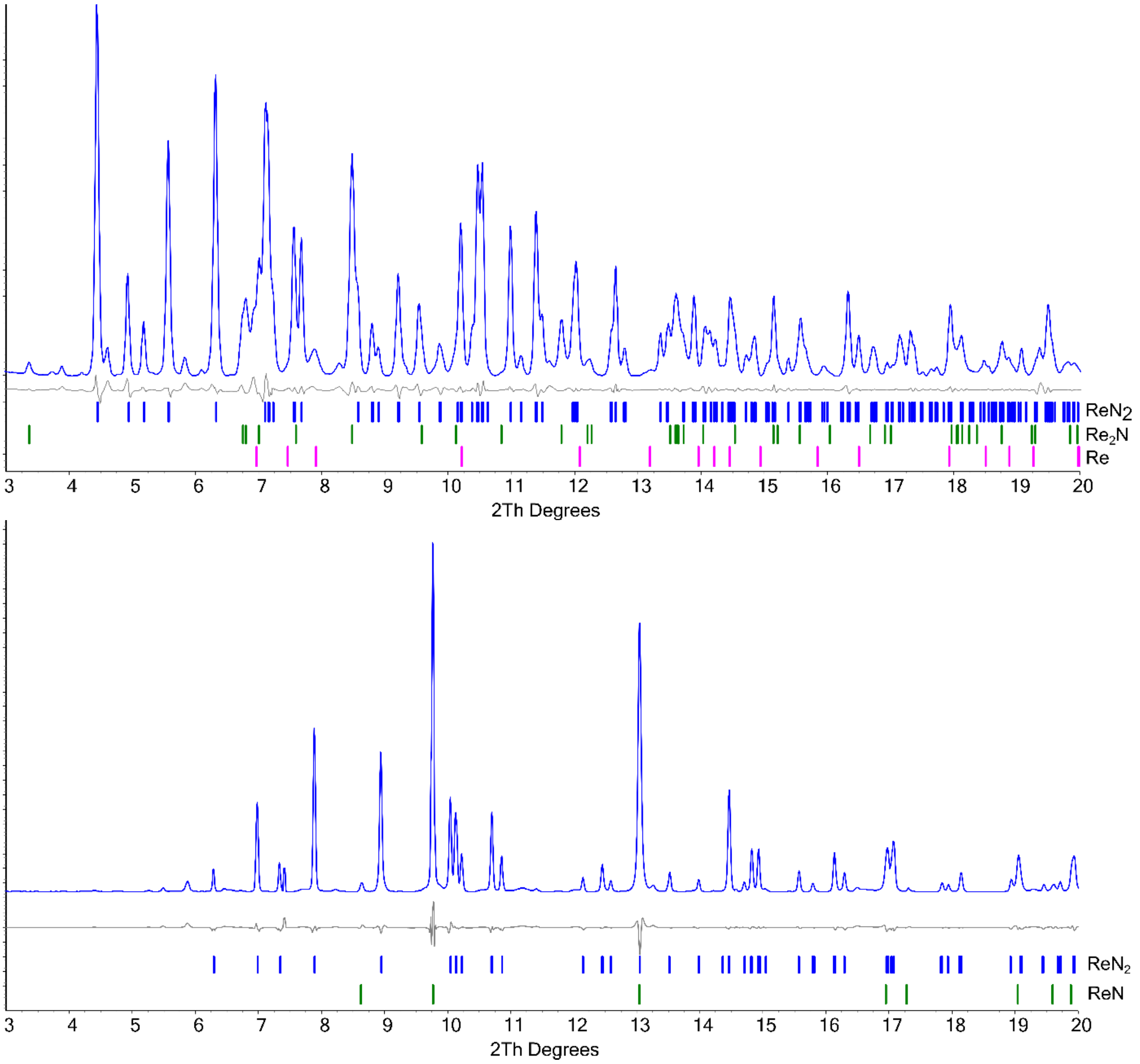

Supplementary Figure 4. Powder diffraction pattern of the sample #1 at ambient pressure (λ = 0.29 and 0.41 Å for upper and lower patterns respectively).

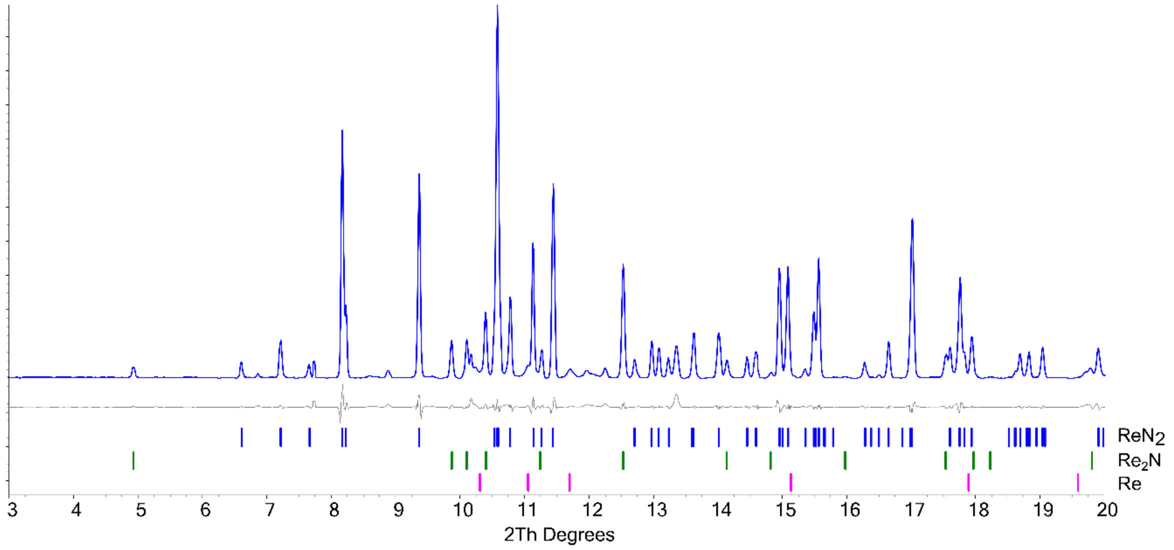

Supplementary Figure 5. Powder diffraction pattern of the sample #3 at 71 GPa (λ = 0.41 Å).

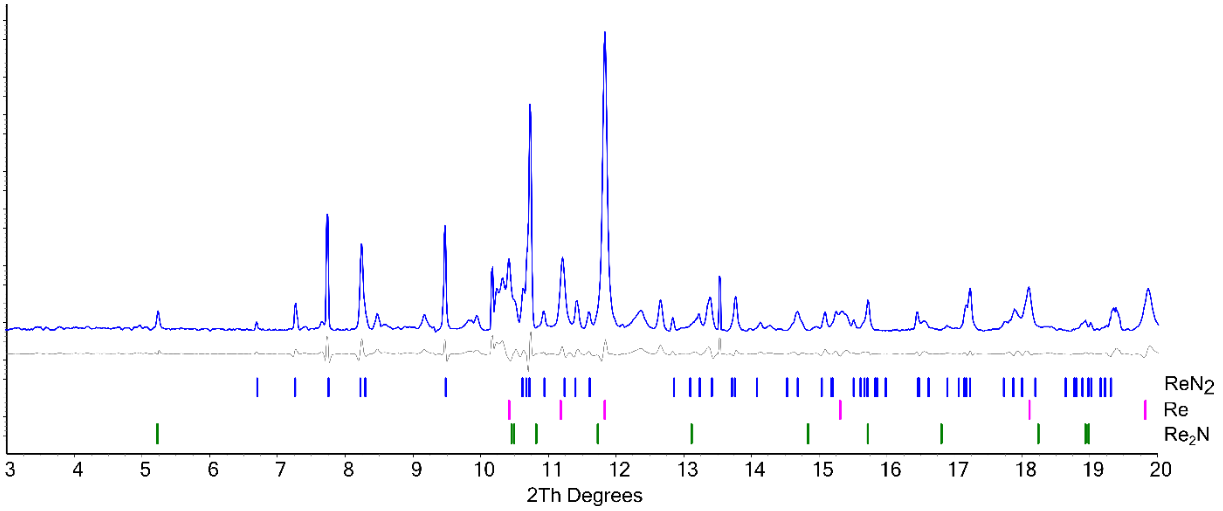

Supplementary Figure 6. Powder diffraction pattern of the sample #4 at 86 GPa (λ = 0.41 Å).

Supplementary Table 5. Experimental lattice parameters of ReN_2 on compression.

Pressure, GPa	*a*, Å	*b*, Å	*c*, Å	*β*, º	*V*, Å^3
0.0001	3.62565(16)	6.4216(2)	4.9478(3)	111.446(4)	107.218(9)
3.37(5)	3.6171(2)	6.4118(4)	4.9234(3)	111.250(5)	106.420(12)
4.10(5)	3.6154(3)	6.4106(6)	4.9161(6)	111.279(8)	106.173(19)
6.95(9)	3.6092(4)	6.3961(7)	4.9050(6)	111.256(8)	105.53(2)
8.97(6)	3.6055(3)	6.3872(8)	4.8974(6)	111.267(8)	105.10(2)
11.18(5)	3.5993(4)	6.3770(8)	4.8822(6)	111.261(11)	104.43(2)
12.79(11)	3.5957(4)	6.3723(8)	4.8715(9)	111.242(12)	104.03(3)
14.74(11)	3.5900(4)	6.3623(8)	4.8617(8)	111.177(12)	103.54(3)
17.97(13)	3.5849(5)	6.3517(8)	4.8523(6)	111.202(11)	103.01(2)
25.67(15)	3.5728(6)	6.3071(10)	4.8226(8)	111.243(14)	101.29(3)
32.11(17)	3.5604(6)	6.2742(10)	4.7992(8)	111.264(14)	99.91(3)
39.37(13)	3.5482(9)	6.2338(13)	4.7735(9)	111.272(17)	98.39(4)
45.44(14)	3.5379(9)	6.1963(10)	4.7503(8)	111.368(15)	96.98(4)

Supplementary Note 4. Choice of solid nitrogen precursors

The reaction between Re and NaN_3 resulted in the main product, which has hexagonal symmetry $P6_3/mmc$ ($a = 2.7715$, $c = 11.2191$ Å). The structure solution and refinement revealed its chemical formula as $NaReN_2$. The structure is based on layered MoS_2-type structure with sodium atoms intercalated between ReN_2 layers (Supplementary Figure 7). Na occupies the crystallographic site 2*a* (0, 0, 0), Re – 2*c* (1/3, 2/3, 1/4), N – 4*f* (1/3, 2/3, 0.6405). It should be noted that the lattice parameters of this phase are very close to those of the ReN_2 phase, that was synthesized by Kawamura *et al.*,[9] with a difference that the lattice parameter *c* of $NaReN_2$ is larger. If we consider the reaction, reported by Kawamura *et al.*:[9]

$$ReCl_5 + 2Li_3N + xNaCl \xrightarrow{7.7\ GPa,\ \ 1200^{\circ}C} ReN_2 + 5LiCl + xNaCl \qquad (1),$$

we can notice two important problems: The equation is not balanced (and cannot be balanced, even if we consider the release of free nitrogen on the right side of the equation) and rhenium is the only element that has changed its oxidation state. We suggest that the real product of the reaction (1) is $LiReN_2$. This explains both issues mentioned above, and is in agreement with shorter *c*-axis than in $NaReN_2$ due to the smaller cation radius of lithium compared to sodium.

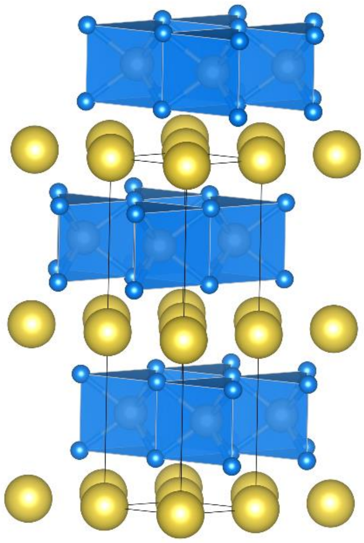

Supplementary Figure 7. The crystal structure of $NaReN_2$ from the synthesis #5 at ~40 GPa. Yellow balls – Na atoms. Blue polyhedra – ReN_6 trigonal prisms. Re occupies the site 2c (1/3, 2/3, ¼), N – 4f (1/3, 2/3, 0.64217), Na – 2*a* (0 0 0).

Sodium azide appeared to be not a suitable source of nitrogen for high-pressure nitridation reaction, but opens a route to ternary nitrides with intercalated alkali metals, which in turn may be important for the development of high-performance electrode materials.[10]

The experiment in LHDAC with NH_4N_3 as a source of nitrogen (Experiment #6, Table 1) resulted in the synthesis of ReN_2 and defect WC-type ReN_x. The unit cell volume of WC-type ReN_x at ambient pressure appeared to be slightly smaller than that in the Experiment#1 [V_{exp1} = 18.42(1) Å, V_{exp6} = 18.19(1) Å^3] and the estimated composition of this compound is $ReN_{0.56}$. The Experiments #6 and 7 show that NH_4N_3 precursor may be successfully used for the synthesis of rhenium nitrides when a solid source of nitrogen is required.

Currently, several methods are used for the synthesis of nitrides in the LVP. One route is a high-pressure solid-state metathesis (HPSSM) reaction between an oxidized metal precursor and a nitride (*e.g.* boron nitride BN or lithium nitride Li_3N).[9,11–14] Recently Lei *et al*. reported a novel synthetic route to rhenium nitride Re_3N:[13,15]

$$3NaReO_4 + 3BN \rightarrow Re_3N + 3O_2 + 3NaBO_2 + 3N_2 \quad (2)$$

Although, the HPSSM method resulted in a number of exciting discoveries, it has a few disadvantages. In case of a simple metathesis reaction, where the oxidation state of elements is not changed it will not be possible to obtain compounds with N-N units. In case of redox reactions, where metal itself serves as an oxidizer of nitrogen, it is hard to create an excess of nitrogen that can prevent decomposition of target nitrogen-rich phases. Furthermore, there are always several side products of such a reaction: *e.g.* the release of free oxygen like in the reaction (3) may influence the reaction in some cases. Schnick *et al.* successfully used controlled decomposition of azides to obtain diazenides BaN_2, SrN_2 and CaN_2 as well as Li_2N_2 in a large volume press.[16,17] This is a much cleaner method than a metathesis reaction, but is demanding, because not all metal azides are readily available and safe to work with. NH_4N_3 appears to be a good choice for the synthesis of binary nitrides of transition metals due to several reasons: It has a high content of nitrogen (93.3 wt. %), and it can serve as an oxidizer, so that the elemental metal can be used for the reaction. Compared with the metal azides, it is relatively safe to work with this material. The dissociation of the excess of NH_4N_3 may create high partial pressure of N_2, which prevents the decomposition of target nitrogen-rich phases (Le Chatelier principle).[18]

Supplementary Note 5. Synthesis of ReN_2 in a multianvil apparatus

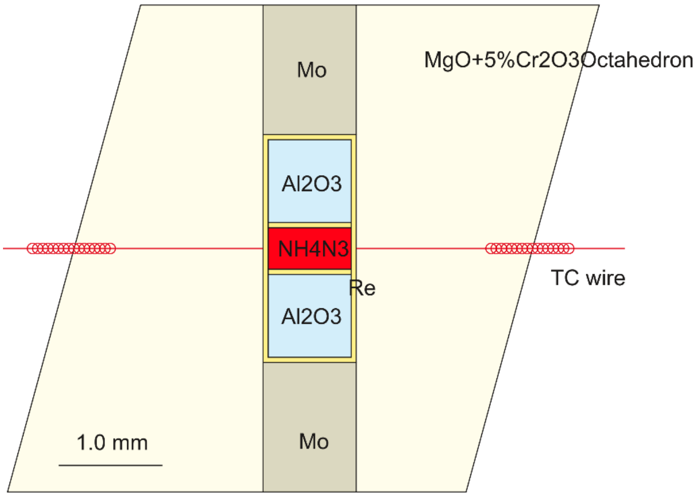

Supplementary Figure 8. Schematic drawing of high-pressure cell assembly for the multianvil synthesis of rhenium nitrides.

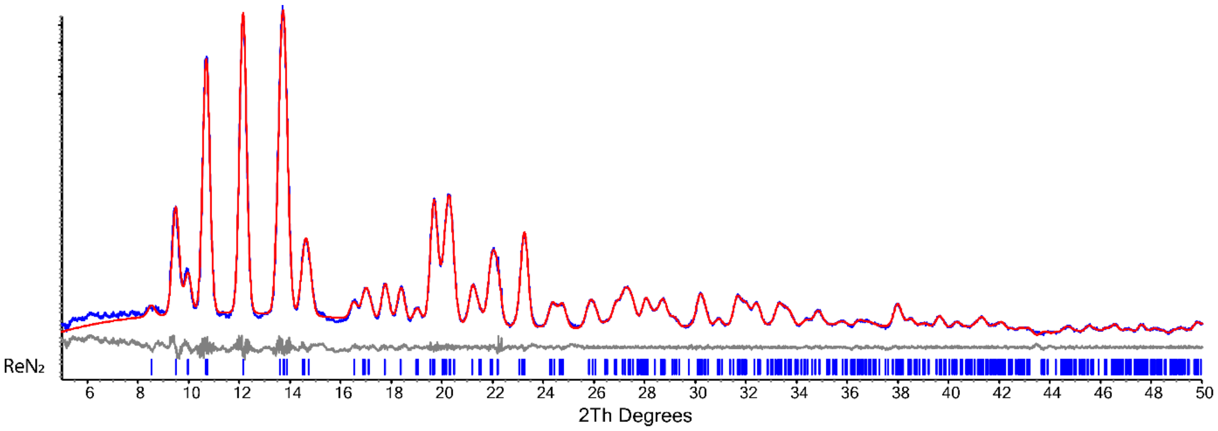

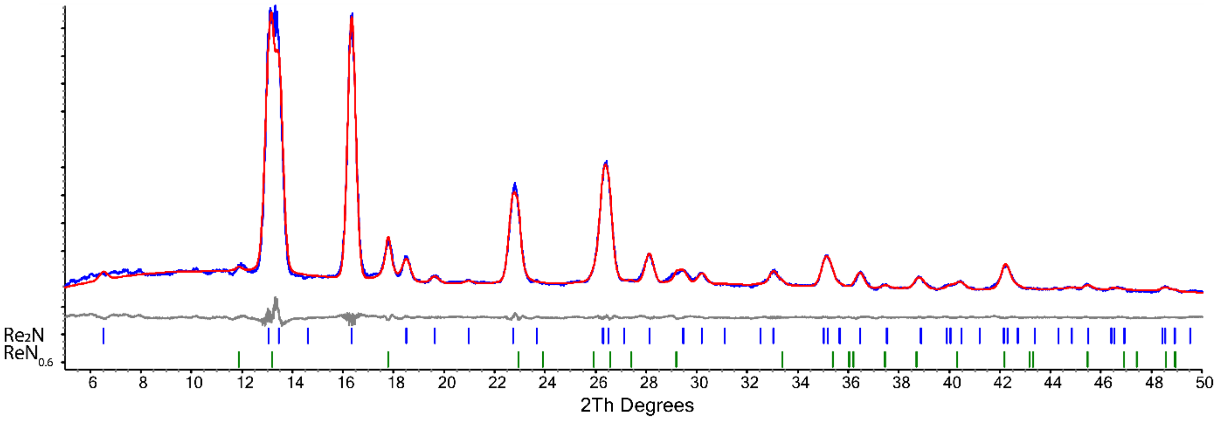

Supplementary Figure 9. Powder diffraction pattern of the samples recovered from the synthesis in the multianvil press (Experiment #7), λ = 0.56 Å (Ag-Kα). ReN_2 and Re_2N phases were characterized by single-crystal X-ray diffraction, while $ReN_{0.6}$ is evidenced only from the powder XRD.

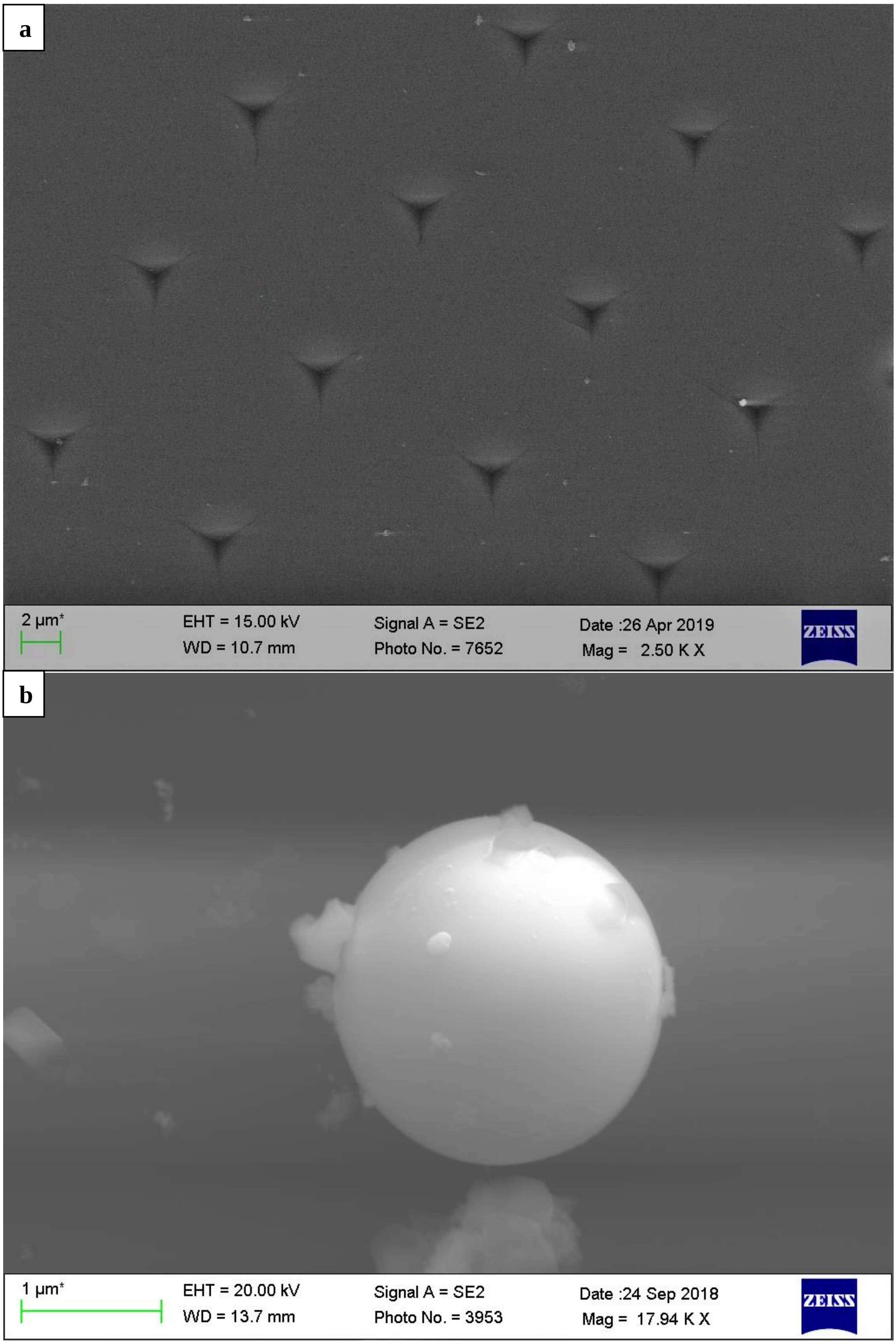

Supplementary Figure 10. (a) SEM image of a ReN_2 sample (Experiment #7) that was used for nanoindentation measurements. (b) SEM image of a ReN_2 sample extracted from the diamond anvil cell (Experiment #6). Images were measured on a ZEISS SEM, Leo Gemini 1530 with a Schottky field emission gun employing an accelerating voltage of 15-20 kV.

Supplementary references

1. Asvini meenaatci, A. T., Rajeswarapalanichamy, R. & Iyakutti, K. First-principles study of electronic structure of transition metal nitride: ReN under normal and high pressure. *Phys. B Condens. Matter* **406,** 3303–3307 (2011).
2. Li, Y.-L. & Zeng, Z. Potential ultra-incompressible material ReN: First-principles prediction. *Solid State Commun.* **149,** 1591–1595 (2009).
3. Dubrovinsky, L., Dubrovinskaia, N., Prakapenka, V. B. & Abakumov, A. M. Implementation of micro-ball nanodiamond anvils for high-pressure studies above 6 Mbar. *Nat. Commun.* **3,** 1163 (2012).
4. Friedrich, A. *et al.* Novel rhenium nitrides. *Phys. Rev. Lett.* **105,** 1–4 (2010).
5. Bykov, M. *et al.* High-Pressure Synthesis of a Nitrogen-Rich Inclusion Compound $ReN_8 \cdot xN_2$ with Conjugated Polymeric Nitrogen Chains. *Angew. Chem., Int. Ed.* **57,** 9048–9053 (2018).
6. Eremets, M. I., Gavriliuk, A. G., Trojan, I. A., Dzivenko, D. A. & Boehler, R. Single-bonded cubic form of nitrogen. *Nat. Mater.* **3,** 558–63 (2004).
7. Zhao, Z. *et al.* Nitrogen concentration driving the hardness of rhenium nitrides. *Sci. Rep.* **4,** 4797 (2014).
8. Zhao, E. & Wu, Z. Electronic and mechanical properties of 5d transition metal mononitrides via first principles. *J. Solid State Chem.* **181,** 2814–2827 (2008).
9. Kawamura, F., Yusa, H. & Taniguchi, T. Synthesis of rhenium nitride crystals with MoS_2 structure. *Appl. Phys. Lett.* **100,** 2–5 (2012).
10. Zhong, Y. *et al.* Transition Metal Carbides and Nitrides in Energy Storage and Conversion. *Adv. Sci.* **3,** 1500286 (2016).
11. Wang, S. M. *et al.* Synthesis, Crystal Structure, and Elastic Properties of Novel Tungsten Nitrides. *Chem. Mater.* **24,** 3023–3028 (2012).
12. Wang, S. *et al.* The Hardest Superconducting Metal Nitride. *Sci. Rep.* **5,** 13733 (2015).
13. Lei, L., Yin, W., Jiang, X., Lin, S. & He, D. Synthetic route to metal nitrides: High-pressure solid-state metathesis reaction. *Inorg. Chem.* **52,** 13356–13362 (2013).
14. Kloß, S. D. & Schnick, W. Nitridophosphates - A Success Story of Nitride Synthesis. *Angew. Chem., Int. Ed.* (2018). doi:10.1002/anie.201812791
15. Jiang, X., Lei, L., Hu, Q., Feng, Z. C. & He, D. High-pressure Raman spectroscopy of Re_3N crystals. *Solid State Commun.* **201,** 107–110 (2015).
16. Schneider, S. B., Frankovsky, R. & Schnick, W. Synthesis of Alkaline Earth Diazenides $M_{AE}N_2$ (M_{AE} = Ca, Sr, Ba) by Controlled Thermal Decomposition of Azides under High Pressure. *Inorg. Chem.* **51,** 2366–2373 (2012).
17. Schneider, S. B., Frankovsky, R. & Schnick, W. High-Pressure Synthesis and Characterization of the Alkali Diazenide Li_2N_2. *Angew. Chem., Int. Ed.* **51,** 1873–1875 (2012).
18. Vogel, S., Buda, A. T. & Schnick, W. United in Nitride: The Highly Condensed Boron Phosphorus Nitride BP_3N_6. *Angew. Chem., Int. Ed.* **57,** 13202–13205 (2018).
