# Supplementary material for: High-pressure synthesis of ultraincompressible hard rhenium nitride pernitride Re2(N2)(N)2 stable at ambient conditions
Source: Nat Commun. 2019 Jul 5;10:2994. doi: 10.1038/s41467-019-10995-3 (PMC6611777; doi:10.1038/s41467-019-10995-3)
Supplement: Supplementary file 3 — Description of Additional Supplementary Files [file 41467_2019_10995_MOESM3_ESM.docx]

**Description of Supplementary Files**

**File Name:** **Supplementary Data 1**

**Description:** Crystallographic information file (cif) for ReN2 at ambient conditions.

**File Name:** **Supplementary Data 2**

**Description:** Structure factor file (fcf) for ReN2 at ambient conditions.

**File Name:** **Supplementary Data 3**

**Description:** The source data underlying Figures 2a-f, and Supplementary Figures 2 a-b.
